# Development, Validation, and Application of Reverse Transcription Real-Time and Droplet Digital PCR Assays for the Detection of the Potyviruses Watermelon Mosaic Virus and Zucchini Yellow Mosaic Virus in Cucurbits

**DOI:** 10.3390/plants12122364

**Published:** 2023-06-19

**Authors:** Marta Luigi, Ariana Manglli, Carla Libia Corrado, Antonio Tiberini, Elisa Costantini, Luca Ferretti, Laura Tomassoli, Sabrina Bertin

**Affiliations:** Research Centre for Plant Protection and Certification, Council for Agricultural Research and Economics, 00156 Rome, Italy

**Keywords:** virus detection, virus quantification, analytical sensitivity, analytical specificity

## Abstract

Among the cucurbit-infecting viruses, watermelon mosaic virus (WMV) and zucchini yellow mosaic virus (ZYMV) (Potyvirus: Potyviridae) are responsible for severe symptoms on cucumber, melon, watermelon, and zucchini cultivations worldwide. In this study, reverse transcription real-time PCR (real-time RT-PCR) and droplet-digital PCR (RT-ddPCR) assays targeting the coat protein (CP) genes of WMV and ZYMV were developed and validated according to the international standards of plant pest diagnosis (EPPO PM 7/98 (5)). First, the diagnostic performance of WMV-CP and ZYMV-CP real-time RT-PCRs was evaluated, and the assays displayed an analytical sensitivity of 10^−5^ and 10^−3^, respectively. The tests also showed an optimal repeatability, reproducibility and analytical specificity, and were reliable for the virus detection in naturally infected samples and across a wide range of cucurbit hosts. Based on these results, the real-time RT-PCR reactions were adapted to set up RT-ddPCR assays. These were the first RT-ddPCR assays aiming at the detection and quantification of WMV and ZYMV and showed a high sensitivity, being able to detect until 9 and 8 copies/µL of WMV or ZYMV, respectively. The RT-ddPCRs allowed the direct estimation of the virus concentrations and opened to a broad range of applications in disease management, such as the evaluation of partial resistance in breeding processes, identification of antagonistic/synergistic events, and studies on the implementation of natural compounds in the integrated management strategies.

## 1. Introduction

Among the limited number of plant families supplying the basis for human diets, the family Cucurbitaceae provides numerous important crops. Starting from their centres of domestication, cucurbit species spread worldwide, facing several constrains such as market demands, necessity for climatic adaptations, and genetic erosion, including the biotic stress challenges. Indeed, each of these crops could be affected by multiple fungal, oomycete, bacterial, and viral diseases as well as insect pests that transmit diseases and cause feeding damage [1]. In particular, cucurbits are highly susceptible to potyviruses (genus *Potyvirus*, family *Potyviridae*) and infections of zucchini yellow mosaic virus (ZYMV), papaya ringspot virus (PRSV), watermelon mosaic virus (WMV), Moroccan watermelon mosaic virus (MWMV) are frequently reported in cucumber, melon, watermelon, and zucchini cultivations [2]. These viruses are mainly transmitted in a non-persistent manner by several aphid species [3], and their management mainly rely on the insect vector control and on the development of tolerant cultivars [2].

Among the potyvirus species, WMV and ZYMV are known to be distributed worldwide because of their adaptability to a wide range of host plants and climatic conditions [4]. The two potyviruses induce similar symptoms, such as mosaic, yellowing, and blister deformations on leaves, and are both responsible for a general stunting of the host plant. Besides this generic symptomatology, some differences can be distinguished between the two viruses: WMV is responsible for vein banding and more or less severe leaf filiformism, whereas ZYMV induces vein clearing on leaves along with external mosaic or necrotic cracks [2,5]. The symptom expression and severity of ZYMV and WMV can depend on several factors, including the virus isolate. Indeed, both ZYMV and WMV genetically evolved in several isolates having different degrees of virulence. WMV isolates were classified into three molecular groups: Group 1, also known as classical (CL), and Group 2 including isolates present in the Mediterranean basin until 2000, when Group 3 emerged (EM) and further evolved into several genetic sub-groups [6,7]. The EM isolates are associated with more severe symptoms and progressively displaced the CL isolates in the Mediterranean basin [8,9,10]. Different genetic groups and subgroups were identified also for ZYMV, based on their geographical distribution: Group A that is present in the Mediterranean basin and is divided into six clusters, and Group B and C that are present in Vietnam and China, and in Poland, respectively [5].

Symptoms of ZYMV and WMV infections are often overlapping because the two potyviruses frequently occur in mixed infection, as reported in several cucurbit crops from the Mediterranean basin to Americas and Asia [11,12,13]. Interactions between co-occurring viruses are known to induce either synergistic or antagonistic effects, namely one or both viruses can benefit or suffer from the presence of each other in terms of viral titre, pathogenicity, and transmission by vectors [14,15]. In the case of ZYMV and WMV, experimental co-infections in zucchini showed a synergistic effect favouring ZYMV, which showed a stronger pathogenic effect than WMV. The presence of ZYMV also negatively affected the replication of WMV, while no similar reciprocal effect was observed. This synergistic interaction led to the prevalence of ZYMV typical symptoms in zucchini, with an increase in yellowing in mixed infections rather than in single WMV infection [16].

Both serological and molecular diagnostic methods are available for the detection of WMV and ZYMV in routine diagnosis, such as enzyme-linked immunosorbent analysis (ELISA) [17], conventional reverse-transcription polymerase chain reaction (RT-PCR) [6,18,19,20], reverse transcription loop-mediated isothermal amplification (RT-LAMP) [21,22], and small RNA sequencing [11]. Most of these methods were not validated for their sensitivity and specificity according to the international requirements for plant pest diagnosis. Moreover, the virus disease management nowadays requires methods not only for the reliable detection of viruses but also for the titre quantification and isolate characterization. Notably, the emergence of novel virus variants as well as the interaction between coinfecting viruses can influence both the virus-virus and virus-plant evolutionary processes, potentially affecting the tolerance of the commercial cultivars [23]. In such cases, a precise quantification of viral copies is needed for evaluating partial resistance in breeding programs and searching for new resistance sources. Moreover, the virus copy quantification in host plants is rising as a prerequisite for implementing disease control strategies with natural compounds in the frame of a sustainable agriculture. Indeed, to better correlate the treatment effect on virus replication and/or key genes in plant response to virus infection, the use quantitative assays are mandatory [24,25].

Real-time PCR and digital PCR (dPCR) represents the best techniques for quantifying the copy of the viruses. Real-time reverse transcription PCR (real-time RT-PCR) protocols already proved to be suitable for both fast and sensitive detection and quantification of potyviruses [23,26]. More recently, the dPCR was shown to further improve the diagnostic and quantification potentials in the field of plant viruses [27,28,29]. Technically, the reaction mixture of dPCR is compartmentalized into thousands of individual reaction vessels using microwell formats, microfluidic chambers, and droplets; each partition contains either zero or few copies of the target and works as individual reaction [30,31]. This compartmentalization improves the technique performances in different ways: the result is acquired by counting the positive *vs*. negative partition helping to reach an absolute quantification of the target without a calibration curve; the reaction is less affected by the presence of inhibitors or background noise; furthermore, the simultaneous run of thousand reactions in each well increase the sensitivity of the method [31].

In this work, a one-step real-time RT-PCR assay was developed for detecting WMV and ZYMV in samples of the main cucurbitaceous crops. The reaction and cycling conditions were optimized, and the protocols were validated according to the EPPO PM 7/98 (5) [32] guidelines, by assessing the analytical sensitivity and specificity, selectivity, repeatability, and reproducibility. Moreover, a RT-dPCR assay using droplet compartmentalization (RT-ddPCR) was further developed based on the real-time RT-PCR reactions. Both real-time RT-PCR and RT-ddPCR assays were then tested for the detection of WMV and ZYMV in naturally infected cucurbit samples.

## 2. Results

### 2.1. Set up of the Real-Time RT-PCR Assays

The virus-specific primers and TaqMan^®^ probes used in this study target the coat protein (CP) genes of WMV and ZYMV. For the detection of WMV, a set of primers and probe already available was tested [33]. This WMV-CP set (Table 1) was based on the conserved CP sequences of WMV-EM isolates which, nowadays, constitute the most widespread genetic group worldwide and, thus, represent the main target of the diagnostic activities. The primers and probe targeting ZYMV were sought on the regions conserved among all the complete CP sequences available in GenBank (NCBI). The ZYMV-CP set (Table 1) was shown to anneal with all the CP sequence included in the *in silico* analysis of inclusivity, suggesting to potentially amplify all the known genetic variants of ZYMV. The exclusivity of both WMV-CP and ZYMV-CP sets was also analysed *in silico* (Blast tool, NCBI) against all the Genbank database. The two sets of primers and probe were, therefore, selected for further analyses.

Preliminary reactions were performed to optimize the real-time RT-PCR conditions. Different annealing temperatures ranging from 55 to 62 °C as well as different primer (100, 300, 600 and 900 nM) and probe (100 and 250 nM) concentrations were tested on the same total RNA (TRNA) extracted from both healthy and WMV/ZYMV-infected samples (Table 2). In all these preliminary reactions, the primer/probe sets were shown to specifically detect the target virus, and no amplification signals were obtained for the negative controls. The WMV-CP primers and probe were shown to produce an exponential amplification curve at the earliest Cq value (12.44) with the annealing temperature set at 60 °C (Figure S1a). This temperature was also within the range that allowed the exponential amplification of ZYMV with the ZYMV-CP set (Cq = 20.65 at 59.5 °C and Cq = 21.69 at 62 °C; Figure S1b). Therefore, 60 °C was chosen as annealing temperature to be used for both the assays. At this condition, the optimal primer and probe concentrations were set to 900 nM and 250 nM, respectively, which resulted in the most efficient amplification of both potyviruses. Indeed, WMV was amplified at a mean Cq value of 14.02, whereas at the other concentrations the mean Cq values ranged between 14.24 to 17.59 with a sensible decrease in relative fluorescence units (RFU); ZYMV was amplified at a mean Cq value of 20.73, whereas at the other concentrations, the mean Cq values ranged between 26.27 and 33.88 (Figure S2).

### 2.2. Validation of the Real-Time RT-PCR Assays

The real-time RT-PCR assays targeting WMV and ZYMV were validated using a panel of cucurbit samples with known phytosanitary status (healthy, single- and double-infected samples) (Table 2).

**Figure 1:**
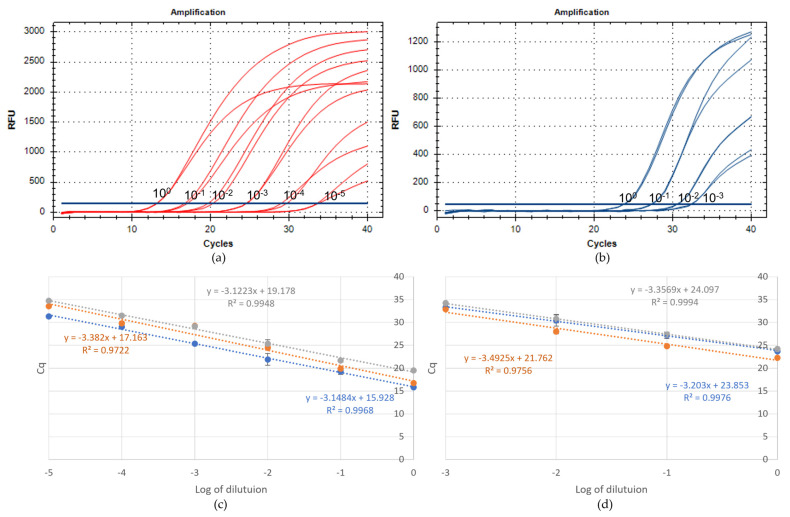
Analytical sensitivity of WMV- and ZYMV-CP real-time RT-PCRs. (**a**) Amplification curves obtained from one out of three tested samples (in technical duplicate) using serial dilutions of WMV TRNA up to 10^−7^ and (**c**) the related standard curves. (**b**) Amplification curves obtained from one out of three tested samples (in technical duplicate) using serial dilutions of ZYMV TRNA up to 10^−5^ and (**d**) the related standard curves. Linear regression formula and coefficient of determination (R^2^) are reported.

TRNAs from healthy samples of *Cucurbita pepo* were spiked with tenfold dilutions of TRNAs from WMV- and ZYMV-infected *C. pepo* samples (three samples per virus) to evaluate the limit of detection (LOD) of the real-time RT-PCR assays for each virus. The analytical sensitivity of WMV and ZYMV was assessed at 10^−5^ (corresponding to mean Cq = 33 for all the three samples) and 10^−3^ dilution (corresponding to mean Cq = 35 for all the three samples), respectively (Figure 1a,b). The serial dilutions were also used to design standard curves and assess geometric efficiency that was calculated from their slopes. Both protocols had optimal values of R^2^ and efficiency (about 105% for WMV and 95% for ZYMV) (Figure 1c,d).

The analytical specificity of real-time RT-PCRs was evaluated considering both inclusivity and exclusivity parameters. Inclusivity of WMV-CP and ZYMV-CP assays was assessed testing five *C. pepo* WMV-infected samples and six ZYMV-infected samples, respectively; two mixed WMV + ZYMV-infected samples were also included in both tests (Table 2). All the target samples resulted positive in each assay. The exclusivity was tested against healthy samples of different cucurbit plant hosts (*C. pepo*, *Citrullus lanatus*, *Cucumis sativus, Cucurbita melo*, and *Cucurbita moschata*) as well as against ten samples infected with some of the most common non-target virus species infecting cucurbits: the potyviruses MWMV and PRSV; *Gammacarmovirus melonis* (former *Melon necrotic spot virus*, MNSV)—genus *Gammacarmovirus*, family *Tombusviridae*; *Cucumber mosaic virus* (CMV)—genus *Cucumovirus*, family *Bromoviridae*; *Cucurbit aphid-borne yellows virus* (CABYV)—genus *Polerovirus*; family *Solemoviridae*; *Squash leaf curl virus* (SLCV), *Watermelon chlorotic stunt virus* (WmCSV), and *Tomato leaf curl New Delhi virus* (ToLCNDV)—genus *Begomovirus;* family *Geminiviridae*; *Cucurbit yellow stunting disorder virus* (CYSDV), and *Beet pseudoyellows virus* (BPYV)—genus *Crinivirus*, family *Closteroviridae* (Table 2). No cross-reactions were obtained in both WMV-CP and ZYMV-CP real-time RT-PCRs. According to the inclusivity and exclusivity results, the analytical specificity of both assays was 100%.

**Figure 2:**
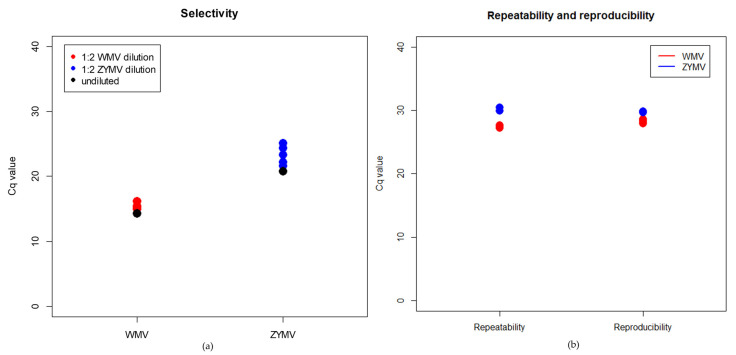
Selectivity, repeatability, and reproducibility of WMV- and ZYMV-CP real-time RT-PCRs. (**a**) Selectivity assay: Cq values obtained in WMV-CP (red dots) and ZYMV-CP (blue dots) real-time RT-PCRs using virus TRNAs diluted in TRNA extracted from five plant species, *C. pepo*, *C. lanatus*, *C. sativus*, *C. melo*, and *C. moschata*; t.q.: undiluted WMV and ZYMV-infected samples (black dot). (**b**) Repeatability and reproducibility assays: Cq values obtained with 10^−4^-fold and 10^−2^-fold diluted WMV (red dots) and ZYMV (blue dots) samples analysed in the same run (repeatability) and after one week and with a different operator (reproducibility).

The possible effect of the plant matrix on the efficiency of the real-time RT-PCRs was evaluated in the selectivity tests by diluting (1:2) the TRNA from WMV- and ZYMV-infected samples in the TRNA extracted from *C. pepo*, *C. lanatus*, *C. sativus*, *C. melo*, and *C. moschata*. The mean Cq values obtained with diluted samples in both WMV (15.33 ± 0.43 SD) and ZYMV amplification (23.32 ± 1.45 SD) was close to the undiluted and water-diluted samples (Figure 2a), suggesting that the amplification efficiency was not affected by different matrices.

The repeatability and reproducibility of the two real-time RT-PCRs were evaluated by analysing in the same run three replicates of WMV- and ZYMV-infected samples with a low concentration of virus. The mean Cq value was 27.42 ± 0.43 SD and 30.08 ± 0.28 SD for WMV- and ZYMV-CP assays, respectively. The same assays were repeated after one week by a different operator, and the mean Cq values were 27.82 ± 0.52 SD and 29.09 ± 0.27 SD for WMV and ZYMV, respectively (Figure 2b). Thus, the repeatability and reproducibility of both WMV- and ZYMV-CP real-time RT-PCRs proved to be 100%.

### 2.3. RT-ddPCR Assays

**Figure 3:**
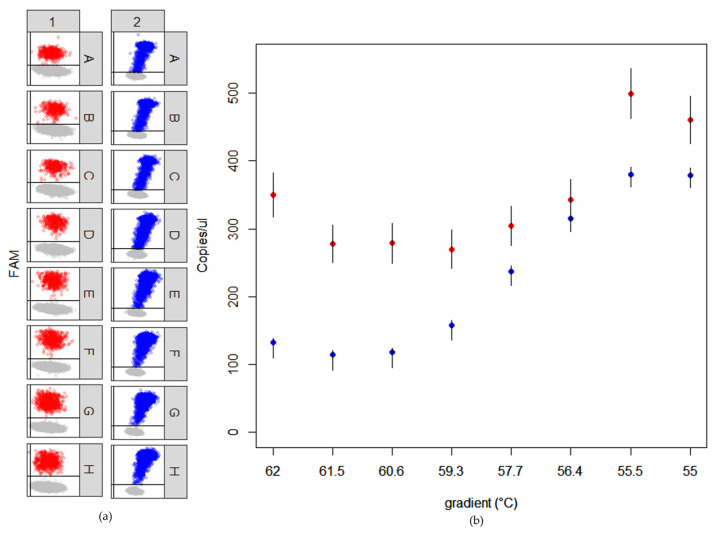
Optimization of annealing temperature in WMV-CP (red) and ZYMV-CP (blue) RT-ddPCRs using isolate 75 for WMV and ZK for ZYMV (Table 1). (**a**) Graphical representation of the separation between positive and negative droplets at the different annealing temperatures (A = 62.0 °C; B = 61.5 °C; C = 60.6 °C; D = 59.3 °C; E = 57.7 °C; F = 56.4 °C; G = 55.5 °C; H = 55.0 °C). (**b**) Number of copies/µL of the target virus detected at each annealing temperature.

The conditions of both WMV-CP and ZYMV-CP RT-ddPCRs were step-by-step optimized using both healthy and WMV/ZYMV-infected samples diluted at 10^−4^ and 10^−3^ in molecular-grade water (Table 2), and considering the optimal separation of the positive and negative droplets. First, the M-MLV reverse transcriptase concentration was tested, maintaining all the other reaction conditions according to the Bio-Rad’s guide. For both WMV-CP and ZYMV-CP assays, the best M-MLV concentration was found to be 20 U for reaction, which produced the largest difference in fluorescence between negative and positive droplets (Figure S3). Then, different annealing temperatures ranging from 55 to 62 °C were tested, and the temperature of 56 °C was chosen as the best compromise in droplet separation and number of copies/µL of target virus detected in both the assays (Figure 3). Additionally, the optimal detection efficiency was obtained by combining 900 nM of each primers and 250 nM of the probe in both WMV-CP and ZYMV-CP RT-ddPCRs (Table 3). In all these preliminary reactions, the primer/probe sets were shown to specifically detect the target virus.

The two RT-ddPCRs were also evaluated for their analytical sensitivity, testing seven tenfold dilutions for each virus (from 10^−1^ to 10^−7^) of three spiked samples obtained mixing TRNAs from *C. pepo* healthy plants with TRNAs from three WMV- and ZYMV-infected *C. pepo* plants. Dilutions from 10^−3^ to 10^−7^ were used to assess the LOD. The last dilution that gave positive results with all the dilutions was 10^−6^ for WMV and 10^−5^ for ZYMV. The two RT-ddPCRs assays were able to detect 9 and 8 copies/µL of WMV or ZYMV, respectively (Table 4).

### 2.4. Test of Naturally Infected Samples

Forty-seven cucurbit samples collected from different Italian regions between 2017 and 2022 were tested for the presence of WMV and ZYMV by real-time RT-PCR (Table 5). Healthy and WMV- and/or ZYMV-infected samples were chosen based on the results of end point RT-PCR assays previously performed.

All the 30 samples expected to be WMV-infected were detected in WMV-CP real-time RT-PCR together with seven samples that tested negative in the previous RT-PCR. These seven samples showed Cq values higher than the known positive samples (>26.50). Among the positive samples, the four ones with a Cq > 29 and the remaining ten negative samples were then assayed using the WMV-CP ddRT-PCR. The four samples were confirmed to be positive in ddRT-PCR, and a further sample (n° 182/2017) was amplified, and showed a very low concentration (11 copies/µL).

The ZYMV-CP real-time RT-PCR amplified only the 16 ZYMV-infected cucurbit samples that were included in the initial batch, whereas none of the putative negative samples produced fluorescent signal. These negative samples, along with a positive sample with a low virus concentration (424/2021; Cq > 30), were further tested in ddRT-PCR. The presence of ZYMV was confirmed in the positive sample, and was detected in two further samples, n° 203/2017 and 51/2020, in 38 and 9 copies/µL, respectively.

## 3. Discussion

The management of plant viral diseases relies on the development of diagnostic techniques that must be rapid, reliable, and adaptable to different matrices. Moreover, methods allowing the quantification of the copy number of the viruses are increasingly requested in virus infection studies. A precise quantification is needed not only in diagnosis for measuring the virus titre and transmission rate but also for evaluating partial resistance in breeding processes, identifying antagonistic/synergistic events, and implementing new control strategies. The real-time and digital PCR techniques are known to meet such requirements, and are increasingly used in routine diagnosis of plant viruses as well as in virus–virus and plant–virus evolutionary studies. In this study, both real-time RT-PCR and RT-ddPCR assays were developed and validated for two economically important cucurbit potyviruses, namely WMV and ZYMV.

First, primers and probe targeting the CP region of both WMV and ZYMV were selected for the development of the virus-specific real-time RT-PCRs. The CP region resulted to be suitable for the specific detection of the two viruses, being conserved within each species but providing sufficient variability to distinguish between potyvirus species. In fact, in silico inclusivity analysis showed that WMV-CP and ZYMV-CP real-time RT-PCRs can amplify all the different genetic subgroups of the predominant WMV-EM and all the known genetic variants of ZYMV, respectively.

Once the reaction conditions were optimized, the WMV-CP and ZYMV-CP real-time RT-PCRs were evaluated and validated according to the current requirements for the implementation of plant pest diagnostic activities in the EU. The sensitivity of the methods was assessed by testing serial dilutions of infected samples. Both WMV-CP and ZYMV-CP reactions showed R^2^ and efficiency close to the *optimum* values, and their LODs were 10^−5^ and 10^−3^, respectively. To date, few real-time RT-PCR assays are available for the detection or quantification of the two viruses and their diagnosis often still relies on ELISA and RT-PCR methods [7,16,23,34]. A small amount of data on sensitivity are therefore available, and the LODs obtained in this study were in line with those reported for the existing methods or even lower [19,20]. This suggests that the developed assays can contribute to improve the current diagnostic potential for the detection of low WMV and ZYMV titers in cucurbit samples.

The WMV-CP and ZYMV-CP real-time RT-PCRs also showed an optimal analytical specificity based on both inclusivity and exclusivity tests. Each assay was able to detect the tested WMV-EM and ZYMV isolates, and was shown to be specific for the related target virus only, since no signals were obtained in the reciprocal reaction and in presence of mixed infections. Moreover, no cross-reactions were observed by testing nine no-target viruses that commonly share the same host plants with WMV and ZYMV, including the potyviruses MWMN and PRSV which are genetically close to the targets. The results of exclusivity tests confirmed the preliminary *in silico* analysis that did not revealed identity of both primer/probe sets with any known sequences.

The repeatability and reproducibility evaluation of WMV-CP and ZYMV-CP real-time RT-PCRs showed a standard deviation between tested samples lower than 1 Cq, confirming that both assays can be used for the detection of WMV and ZYMV with a high degree of confidence. As these results were obtained independently on different days and by different operators, the validation data can be considered robust.

Although the development and optimization of the WMV-CP and ZYMV-CP real-time RT-PCRs were firstly based on infected samples of *C. pepo*, both assays could detect the viruses also when their RNA was spiked in TRNA extracted from other cucurbit host plants, such as *C. lanatus*, *C. sativus*, *C. melo*, and *C. moschata*. Indeed, the selectivity tests showed that no cross-reactions occurred in none of this matrix, and the obtained Cq values were closed to those of the non-spiked samples for both viruses. This suggests that the two assays allow the reliable and efficient detection of WMV and ZYMV across the broad range of their cucurbit hosts.

The optimized WMV-CP and ZYMV-CP real-time RT-PCRs were then transferred to the RT-ddPCR format. The analytical sensitivity of RT-ddPCR was up to 10- and 10^2^-fold higher than RT-qPCR for WMV and ZYMV, respectively. Thus, WMV-CP and ZYMV-CP RT-ddPCR were confirmed to be more sensitive than the corresponding real-time RT-PCRs. In addition to the increased sensitivity, the RT-ddPCR also offers the advantage to directly estimate the concentration of viruses, by measuring the absolute number of viral RNA copies in the TRNA sample without a standard curve as calibrator. This results in a time-saving, avoiding the need to perform several additional steps such as *in vitro* transcription assays, required in case of RNA viruses. In this case, the LOD of each assay was estimated to correspond to 9 and 8 copies/µL of WMV and ZYMV, respectively. These estimates highlight the great potential of the ddPCR to detect even very low viral loads and make the technique highly adaptable to a broad range of studies, both applicative and evolutive. Indeed, the chance to measure the number of viral copies is essential in breeding programs for monitoring the evolution of virus genetic variants and their ability to break resistances of the selected tolerant varieties as well as for searching new potential resistance resources. The virus quantification is even more important for co-infecting viruses, such as WMV and ZYMV, since it gives the opportunity to follow their co-evolution in terms of synergistic or antagonistic events and to predict the possible prevalence of one of them during disease evolution. Finally, the chance to quantify quickly and in an absolute manner the virus titre in the plant tissues makes the RT-ddPCR particularly suitable for the evaluation of the efficacy of natural compounds against viral pathogens, allowing to correlate virus titre and plant treatment. This results in great help for the implementation of natural compounds in integrated management strategies.

The good performance of WMV-CP/ZYMV-CP real-time RT-PCRs and RT-ddPCRs observed during the validation and optimization processes were confirmed by testing naturally infected samples. The panel of tested samples included all the possible combinations of infection *status* assessed by end-point RT-PCR: healthy WMV- and ZYMV-singly infected and mixed infected samples in different cucurbit host species. The developed tests allowed the detection of the target viruses not only in all the expected positive samples but also in samples that tested negative in end point RT-PCR, according to their higher analytical sensitivity. The RT-ddPCR was the most sensitive method, being able to detect the target viruses even at very low concentrations, i.e., 11 copies/µL for WMV and 9 copies/µL for ZYMV. The highest performance of the RT-ddPCRs was also due to the fact that the technique is less prone to PCR inhibitors compared to the real-time RT-PCR and can, therefore, improve the detection sensitivity in TRNA extracted from field samples.

In conclusion, real-time RT-PCR and RT-ddPCR tests targeting WMV and ZYMV were developed in this study. Both assays were reliable and accurate across a wide range of cucurbit hosts. The real-time RT-PCRs were validated according to the current guidelines for plant pest diagnosis (EPPO PM 7/98 (5) [32], providing the first detection tests for WMV and ZYMV that satisfy such international requirements of validation. A reliable and validated test represents an important diagnostic tool in pest management programmes, both for monitoring and control activities. The RT-ddPCR methods described in this study represent the first digital PCR tests that were developed for these potyviruses, and the obtained LODs proved that the assays can detect the targets even in case of low virus titres. This high sensitivity opens to a broad range of applications in disease management beyond the sole virus detection.

## 4. Materials and Methods

### 4.1. Plant Material and RNA Extraction

The tests for the WMV and ZYMV detection were developed and validated using cucurbit samples (*C. pepo*, *C. melo*, *C. lanatus*, *C. sativus*, and *C. moschata*) listed in Table 2 and available at the collection of the Council for Agricultural Research and Economics, Research Centre for Plant Protection and Certification (CREA-DC), except for the samples PC-1271 and PV-0830, which were purchased from DMSZ (Leibniz Institute, Braunschweig, Germany). The sample list included healthy samples as well as samples infected with target and non-target viruses both in single and mixed infection. The samples were conserved as dried or fresh leaf tissues.

Leaf tissues were ground in phosphate buffer 1 M, pH 7,2 (1:5 w:v) and 100 µL of solution were used for the extraction of the total RNA using Total RNA Tissues and Cells kit (Danagen-Bioted S.L., Badalona, Spain) following the manufacturer’s instructions.

### 4.2. Primer and Probe Design

For the detection of WMV, the primers and probes tested in this study were already available in the literature (Table 1, [33]). For the detection of ZYMV, all the available complete sequences of the CP gene were retrieved from GenBank (NCBI) and aligned using Mega v.11 software [35]. The conserved regions among isolates were used to construct primers and probes using PrimerExpress 3 tool (Thermo Fisher Scientific, Waltham, MA, USA) and applying the selection criteria suggested by the ddPCR Application Guide (Bio-Rad, Hercules, CA, USA, bulletin 6407). Primer and probe sequences were manually adjusted when needed. Several potential primers and TaqMan^®^ probes were then tested for their inclusivity and exclusivity in silico using the Blast tool from National Center for Biotechnology Information (NCBI, https://blast.ncbi.nlm.nih.gov/Blast.cgi, accessed on 5 May 2023). The selected primers and probes are listed in Table 1.

### 4.3. Real-Time RT-PCR Set Up

The selected TaqMan^®^ probe and primer sets were synthesized by Eurofins genomics (Kolhn, Germany). Preliminary assays were performed to set up the real-time RT-PCR conditions, by including one negative, two WMV-, and two ZYMV-infected samples (Table 2). These samples were tested at eight annealing temperatures ranging from 55 to 62 °C using primers and probes concentrated at 600 nM and 100 nM, respectively, as suggested by MasterMix instructions (TaqMan^®^ RNA-to-Ct™ 1-Step Kit, Thermo Fisher Scientific). Later, different primer (100, 300, 600, and 900 nM) and probe (100 and 250 nM) concentrations were also evaluated. Once the amplification conditions were optimized, reactions were carried out for both the WMV and ZYMV assays in 10 µL reaction volume containing 5 µL of 2× Mastermix, 0.25 µL of 40× RT enzyme (both from TaqMan^®^ RNA-to-Ct™ 1-Step Kit, Thermo Fisher Scientific), 900 nM of each primer, 250 nM of labelled TaqMan^®^ probe, and 1 µL of template TRNA. The optimized one step real-time RT-PCR cycling conditions included a RT step at 48 °C for 30 min, an initial denaturation step at 95 °C for 10 min, and 40 cycles of denaturation and annealing/elongation steps at 95 °C for 15 s and 60 °C for 1 min, respectively. Analyses were performed using the thermocycler Bio-Rad CFX Maestro v2.2.

The same real-time RT-PCR protocol, including the reverse transcription step, was also applied in exclusivity assays to detect non-target viruses having a DNA genome, since this step did not affect the possibility to amplify the DNA.

### 4.4. Validation of the Real-Time RT-PCR Assays

The real-time RT-PCRs for WMV and ZYMV detection were validated according to the EPPO PM 7/98 (5) [32], by assessing the analytical sensitivity and specificity, selectivity, repeatability, and reproducibility.

#### 4.4.1. Analytical Sensitivity

Three samples infected with each virus were ten-fold serially diluted in healthy plant tissue extracts from 10^−1^ up to 10^−7^ for WMV and 10^−5^ for ZYMV (Table 2). The maximum dilution of the three sample extracts that gave a positive result was established as LOD for each virus. In addition, efficiency of each assay was calculated by interpolating the results obtained from the analysis of the LOD Cq values, using the following equation that determines the efficiency (E) from the slope of the linear regression model [36]:

$$E\left(\%\right)=\left(10^{1-slope}-1\right)\times 100$$

The linear correlation coefficient (R^2^) was also reported.

#### 4.4.2. Analytical Specificity

Inclusivity and exclusivity of the real-time RT-PCRs were assessed firstly *in silico* by questioning the NCBI database, and then, by analysing five WMV-infected samples, six ZYMV-infected samples, and two mixed WMV-ZYMV samples (Table 2). Eleven cucurbit samples infected with non-target viruses were also tested to further ascertain the exclusivity of both real-time RT-PCR assays (Table 2).

#### 4.4.3. Selectivity

TRNA from zucchini plants infected with WMV and ZYMV were diluted 1:2 in TRNA extracted from five healthy samples belonging to different cucurbit species, namely *C. pepo*, *C. lanatus*, *C. sativus*, *C. melo*, and *C. moschata* (Table 2), to determine if the matrix could affect the performance of the real-time RT-PCRs. The Cq values obtained from each WMV and ZYMV dilution were then compared with those obtained for the non-diluted target TRNA extracts and with the target extracts diluted 1:2 in molecular grade water.

#### 4.4.4. Repeatability and Reproducibility

The repeatability of the two real-time RT-PCRs was evaluated by analysing in the same run three replicates of WMV and ZYMV TRNA from one sample for each virus (Table 2). The TRNA was 10^−4^- and 10^−2^-fold diluted for the WMV and ZYMV samples, respectively. The same analyses were repeated at different days and by different operators to assess the reproducibility.

### 4.5. RT-ddPCR Set Up

The one-step RT-ddPCR assays were performed in the QX200TM Droplet Digital PCR system (Bio-Rad, Hercules, CA, USA), according to the manufacturer’s instructions and using the same primers and probes designed for the real-time RT-PCR assays. Preliminary runs with one negative, two WMV-, and two ZYMV-infected samples were performed to set up the RT-ddPCR conditions (Table 2). The tested conditions included 20, 50, and 200 U per reaction of M-MLV (Thermo Fisher Scientific), different annealing temperature ranging from 55 to 62 °C, and different primers (450 and 900 nM) and probe (125 and 250 nM) concentrations. Once the amplification conditions were optimized, both RT-ddPCRs targeting WMV and ZYMV were carried out in a 20 µL reaction volume containing 10 µL of 2× digital PCR supermix for probes (no dUTP) (Bio-Rad), 0.9 µM of each primer, 0.25 µM of TaqMan^®^ probe, DTT 10 mM, 20 U M-MLV, and 2 μL of TRNA. Then, the reaction mixture was placed in a well in the 8-well droplet generator cartridge with 70 μL of droplet generator oil. Droplets were produced using a droplet generator (Bio-Rad) and about 40 μL of the resulting emulsion transferred to a PCR plate, which was heat-sealed using a PX1TM PCR Plate Sealer (Bio-Rad) and placed in a C1000 Thermal Cycler (Bio-Rad). The optimized one step RT-ddPCR cycling conditions (temperature ramp rate, 2 °C/s) included a RT step at 42 °C for 1 h, an initial denaturation step at 95 °C for 10 min, followed by 45 cycles of denaturation and annealing/elongation at 94 °C for 30 s and at 56 °C for 1 min, respectively, followed by last steps at 98 °C for 10 min and 72 °C for 5 min.

The optimized protocols were tested for their analytical sensitivity. Three samples infected with each virus were ten-fold serially diluted in healthy plant tissue extracts from 10^−1^ up to 10^−7^ for WMV and up to 10^−5^ for ZYMV (Table 2). The maximum dilution of the three sample extracts giving a positive result was established as the LOD of WMV-CP and ZYMV-CP RT-ddPCR assays.

### 4.6. Test of Naturally Infected Samples

Both the real-time RT-PCR and RT-ddPCR methods were tested on naturally infected cucurbit samples following the optimized protocols described above. A total of 47 samples of *C. melo*, *C. pepo*, *C. lanatus*, and *C. maxima* collected at different Italian locations between 2017 and 2022 were selected among those that resulted to be healthy or infected by WMV or ZYMV in previous end point RT-PCR assays according to Bertin et al., 2020, and Mohamed et al., 2014 [10,18] (Table 5). First, all the samples were analysed by both WMV-CP and ZYMV-CP real-time RT-PCRs. Then, the samples still resulting negative and the positive samples with high Cq values (>29) were analysed by RT-ddPCR.

### 4.7. Data Analysis

Data from RT-qPCRs and RT-ddPCRs were analyzed using software R version 4.1.1 [37]. RT-ddPCR graphs were obtained using the ddPCR R package version 1.15, a tool that allows the analysis and visualization of Droplet Digital PCR data in R [38].

## Figures and Tables

**Table 1:** Primer and probe sequences for the amplification of WMV- and ZYMV-CP and their positions on the WMV and ZYMV reference genomes retrieved from GenBank.

Name	Sequence (5′-3′)	Position (Sequence ID)	Reference
WMV-CP Probe	FAM-CCAACAAAAGCTGGCACAGTCAGCAA-BH1	9061-9086 (MN_296125.1)	[33]
WMV-CP F	TGGGCAGGGTAGCAAGGA	9042-9059 (MN_296125.1)	
WMV-CP R	CCTTTTGATCCAACGTTCACATC	9088-9110 (MN_296125.1)	
ZYMV-CP Probe	FAM-AGCCAACTGTGGCAGATGCTGGAGCT-BH1	8552-8573 (NC_003224.1)	This study
ZYMV-CP F	CCTACAAGCCCTCCATCAAG	8484-8503 (NC_003224.1)	
ZYMV-CP R	ACTGTTTTCTCACCTGAGCC	8632-8651 (NC_003224.1)	

**Table 2:** List of the samples used in the set-up and validation of the real-time RT-PCR and RT-ddPCR assays for the detection of WMV (W) and ZYMV (Z) or both (WZ). The panel includes cucurbit samples with known phytosanitary status: healthy and infected with target and non-target viruses in single or mixed infection.

			*Real-Time RT-PCR*						*RT-ddPCR*	
*Phytosanitary* *Status*	Sample ID	Host	Test Set-Up	Analytical Sensitivity	Analytical Specificity—Inclusivity	Analytical Specificity—Exclusivity	Selectivity	Repeatability/ Reproducibility	Test Set-Up	Analytical Sensitivity
*WMV*	36/12	*C. pepo*		W	W	Z		W		W
*WMV*	75/20	*C. pepo*	W	W	W	Z	W	W	W	W
*WMV*	136/20	*C. pepo*	W	W	W	Z		W	W	W
*WMV*	163/17	*C. pepo*			W	Z				
*WMV + CABYV*	86/18	*C. melo*			W	Z				
*WMV + ZYMV*	191/17	*C. melo*			WZ					
*WMV + ZYMV*	202/17	*C. melo*			WZ					
*ZYMV*	23/12	*C. pepo*			Z	W				
*ZYMV*	32Syn/12	*C. pepo*			Z	W				
*ZYMV*	1ZB	*C. pepo*	Z	Z	Z	W	Z	Z	Z	Z
*ZYMV*	ZK	*C. pepo*	Z	Z	Z	W		Z	Z	Z
*ZYMV*	ZB10.2	*C. pepo*		Z	Z	W		Z		Z
*ZYMV*	31Syn/12	*C. pepo*			Z	W				
*Healthy*	-	*C. pepo*	WZ	WZ		WZ	WZ	WZ	WZ	WZ
*Healthy*	-	*C. lanatus*				WZ	WZ			
*Healthy*	-	*C. sativus*				WZ	WZ			
*Healthy*	-	*C. melo*				WZ	WZ			
*Healthy*	-	*C. moschata*				WZ	WZ			
*MNSV*	MNSV	*C. lanatus*				WZ				
*CMV*	104/16	*C. pepo*				WZ				
*MWMV + PRSV*	144/19	*C. pepo*				WZ				
*SLCV*	PC-1271	unknown				WZ				
*WmCSV*	PV-0830	unknown				WZ				
*CABYV*	153/17	*C. melo*				WZ				
*ToLCNDV*	198/18	*C. pepo*				WZ				
*PRSV*	45Syn/12	*C. pepo*				WZ				
*CYSDV*	178/17	*C. pepo*				WZ				
*BYPV*	60/16	*C. melo*				WZ				

**Table 3:** Number of copies/µL of the target virus obtained with different combinations of primer and probe concentrations in WMV-CP and ZYMV-CP RT-ddPCRs. The highest values corresponding to the most efficient combinations were bolded. NT—not tested.

		Primers			
		WMV		ZYMV	
		450 nM	900 nM	450 nM	900 nM
**Probe**	125 nM	317 copies/µL	NT	178 copies/µL	NT
	250 nM	241 copies/µL	**343 copies/µL**	219 copies/µL	**315 copies/µL**

**Table 4:** Number of copies/µL of the target virus obtained in WMV-CP and ZYMV-CP RT-ddPCRs at the different dilutions tested for the three samples used.

		Dilutions				
		10^−7^	10^−6^	10^−5^	10^−4^	10^−3^
Samples	WMV 1	49	120	450	3880	28,900
	WMV 2	9	29	54	1790	21,240
	WMV 3	-	12.4	40	395	2880
	ZYMV 1	-	-	8	35	559
	ZYMV 2	-	-	22	131	610
	ZYMV 3	-	-	47	229	2160

**Table 5:** Detection of WMV and ZYMV in 47 samples of *C. melo*, *C. pepo*, *C. lanatus*, and *C. maxima* collected in the field at different locations between 2017 and 2022, and analysed by end-point RT-PCR, real-time RT-PCR, and RT-ddPCR. Non-concordant results between RT-PCR and real-time RT-PCR, and between real-time RT-PCR and RT-ddPCR are in bold. NT: not tested.

SAMPLES			WMV			ZYMV		
**ID/Year**	**Host**	Region/Location	RT-PCR	Real-Time RT-PCR (Cq)	RT-ddPCR (Copies/µL)	RT-PCR	Real-Time RT-PCR (Cq)	RT-ddPCR (Copies/µL)
102/2017	*C. melo*	Sardinia/Arborea	+	13.37	NT	-	-	-
105/2017	*C. melo*	Sardinia/Serramanna	+	12.13	NT	-	-	-
106/2017	*C. melo*	Sardinia/Uta	-	-	-	-	-	-
113/2017	*C. melo*	Sardinia/Arborea	-	**32.82**	64	-	-	-
130/2017	*C. melo*	Sardinia/Arborea	-	**27.71**	NT	-	-	-
136/2017	*C. melo*	Sardinia/Arborea	+	14.70	NT	-	-	-
137/2017	*C. melo*	Sardinia/Arborea	+	17.03	NT	-	-	-
139/2017	*C. melo*	Sardinia/Arborea	-	**30.85**	375	-	-	-
163/2017	*C. melo*	Sardinia/Arborea	+	15.98	NT	-	-	-
178/2017	*C. melo*	Sardinia/Uta	-	-	-	-	-	-
182/2017	*C. melo*	Sardinia/Uta	-	-	**11**	-	-	-
189/2017	*C. melo*	Sardinia/Serramanna	+	22.07	NT	+	23.98	NT
190/2017	*C. melo*	Sardinia/Serramanna	+	22.77	NT	+	25.49	NT
191/2017	*C. melo*	Sardinia/Serramanna	-	**29.48**	87	-	-	-
192/2017	*C. melo*	Sardinia/Serramanna	+	20.11	NT	+	27.05	NT
201/2017	*C. melo*	Sardinia/Serramanna	+	18.57	NT	+	25.30	NT
203/2017	*C. melo*	Sardinia/Serramanna	-	**30.76**	240	-	-	**38**
143/2019	*C.pepo*	Latium/Fondi	-	-	-	-	-	-
147/2019	*C. pepo*	Latium/Fondi	-	-	-	-	-	-
148/2019	*C. pepo*	Latium/Fondi	-	-	-	-	-	-
51/2020	*C. pepo*	Calabria/L. Terme	+	20.32	NT	-	-	**9**
52/2020	*C. pepo*	Calabria/L. Terme	+	14.46	NT	-	-	-
53/2020	*C. pepo*	Calabria/Cosenza	-	**27.50**	NT	-	-	-
60/2020	*C. pepo*	E. Romagna/Cesena	-	**26.89**	NT	-	-	-
61/2020	*C. pepo*	E. Romagna/Cesena	+	23.37	NT	+	24.02	NT
62/2020	*C. pepo*	E. Romagna/Cesena	+	20.58	NT	-	-	-
63/2020	*C. pepo*	E. Romagna/Cesena	+	24.87	NT	+	23.72	NT
64/2020	*C. pepo*	E. Romagna/Cesena	-	-	-	+	22.71	NT
364/2021	*C. pepo*	Veneto/Verona	+	18.04	NT	-	-	-
365/2021	*C. pepo*	Veneto/Verona	+	18.73	NT	-	-	-
366/2021	*C. pepo*	Veneto/Verona	+	18.07	NT	-	-	-
367/2021	*C. pepo*	Veneto/Verona	+	23.35	NT	-	-	-
368/2021	*C. pepo*	Veneto/Verona	+	26.12	NT	-	-	-
369/2021	*C. lanatus*	Marche/Pesaro-Urbino	+	16.13	NT	-	-	-
370/2021	*C. pepo*	Veneto/Verona	+	15.15	NT	-	-	-
407/2021	*C. pepo*	Veneto/Rovigo	+	20.33	NT	-	-	-
423/2021	*C. pepo*	Piedmont/Torino	+	18.74	NT	+	27.95	NT
424/2021	*C. pepo*	Piedmont/Torino	+	19.35	NT	+	30.53	400
7/2022	*C. maxima*	Latium	-	-	-	-	-	-
8/2022	*C. maxima*	Latium	-	-	-	-	-	**9**
37/2022	*C. pepo*	Latium/Latina	+	18.70	NT	+	23.50	NT
38/2022	*C. pepo*	Latium/Latina	-	-	-	+	24.61	NT
39/2022	*C. pepo*	Latium/Latina	+	13.76	NT	+	22.87	NT
40/2022	*C. pepo*	Latium/Latina	+	19.04	NT	+	25.71	NT
41/2022	*C. pepo*	Latium/Latina	+	13.78	NT	+	23.71	NT
42/2022	*C. pepo*	Latium/Latina	+	18.07	NT	+	24.06	NT
43/2022	*C. pepo*	Latium/Latina	+	13.33	NT	+	22.36	NT

## Data Availability

Not applicable.

## Supplementary Materials

The following supporting information can be downloaded at: https://www.mdpi.com/article/10.3390/plants12122364/s1. Figure S1. Optimization of the real-time RT-PCR annealing temperature (using 600 nM of pri-mers and 100 nM of probes); Figure S2. Optimization of the real-time RT-PCR primer and probe concentrations; Figure S3. Optimization of M-MLV concentration (20, 50 and 200 U per reaction) in WMV-CP (red) and ZYMV-CP (blue) RT-ddPCRs.
