# Supplementary material for: Development, Validation, and Application of Reverse Transcription Real-Time and Droplet Digital PCR Assays for the Detection of the Potyviruses Watermelon Mosaic Virus and Zucchini Yellow Mosaic Virus in Cucurbits
Source: Plants (Basel). 2023 Jun 19;12(12):2364. doi: 10.3390/plants12122364 (PMC10302964; doi:10.3390/plants12122364)
Supplement: Supplementary file 1 [file plants-12-02364-s001.zip › plants-2414204-supplement.pdf]

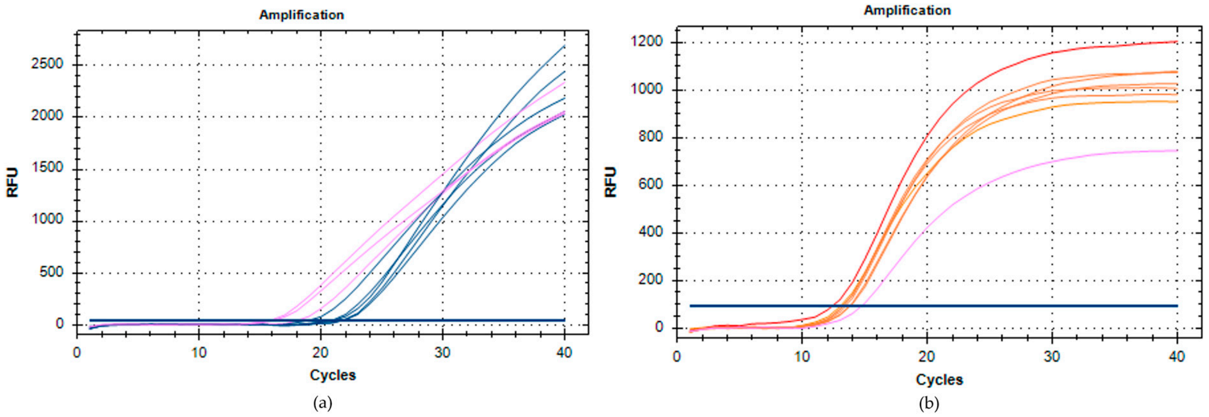

Figure S1. Optimization of the real-time RT-PCR annealing temperature (using 600 nM of primers and 100 nM of probes). (**a**) Eight amplification curves obtained in WMV-CP real-time RT-PCR with different annealing temperatures: red curve, 60°C; orange curves, 56-61°C; pink curve, 55°C. (**b**) Eight amplification curves obtained in ZYMV-CP real-time RT-PCR with different annealing temperatures: non-exponential pink curves, 55-57°C; exponential blue curves, 57.8-62 °C.

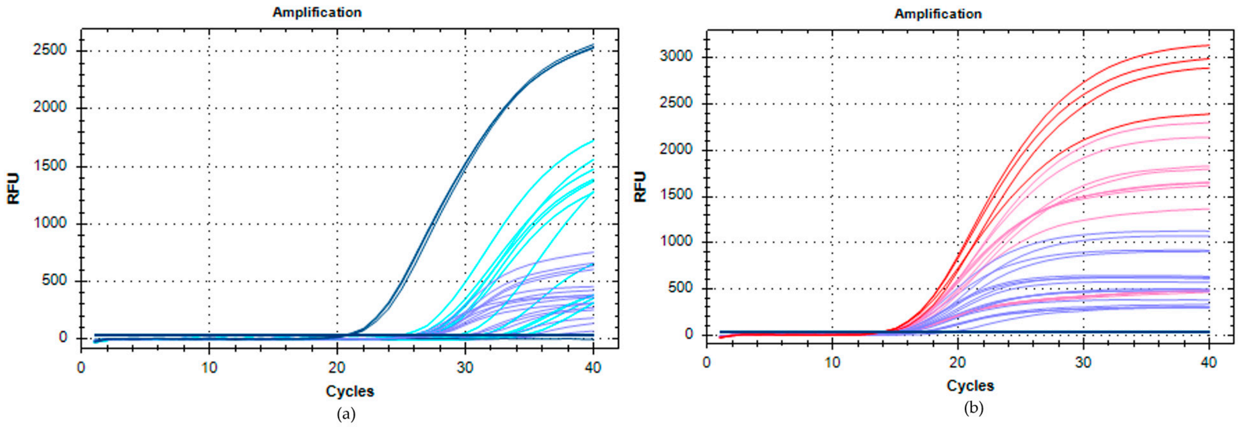

Figure S2. Optimization of the real-time RT-PCR primer and probe concentrations. (a) Amplification curves obtained in WMV-CP real-time RT-PCR with different primer and probe concentrations: red curves, combination of 900 nM of each primer and 250 nM of probe; pink curves, combination of 100, 300, 600 nM of each primer and 250 nM of probe; purple curves, combination of 100, 300, 600, 900 nM of each primer and 100 nM of probe. (b) Amplification curves obtained in ZYMV-CP real-time RT-PCR with different primer and probe concentrations: blue curves, combination of 900 nM of each primer and 250 nM of probe; light-blue curves, combination of 100, 300, 600 nM of each primer and 250 nM of probe; purple curves, combination of 100, 300, 600, 900 nM of each primer and 100 nM of probe.

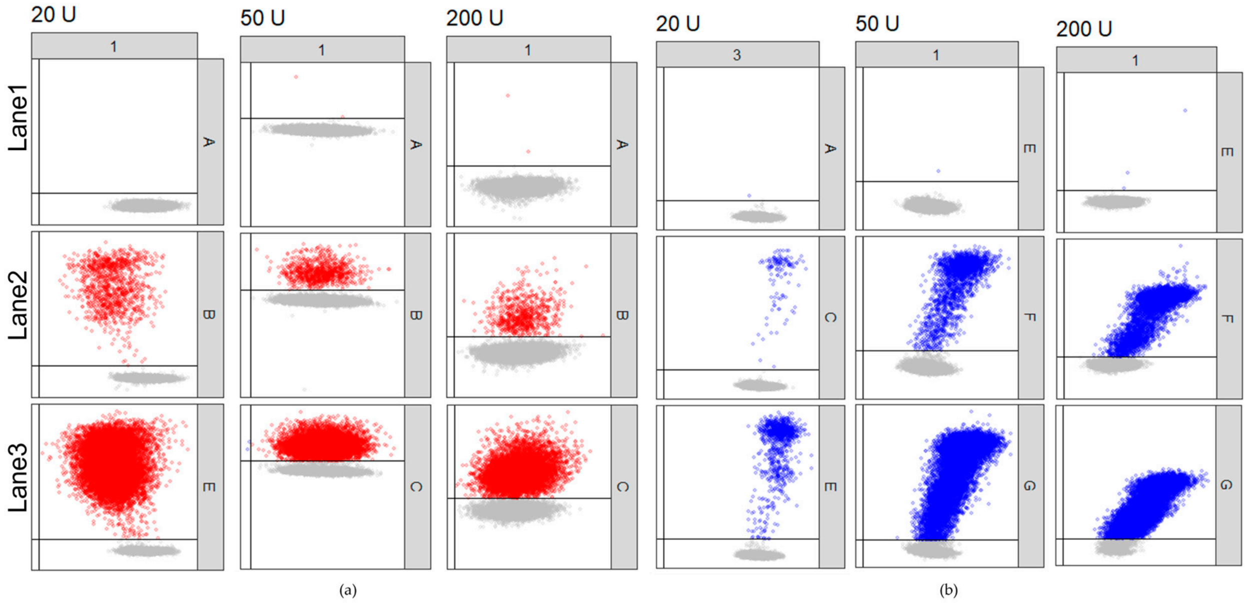

Figure S3. Optimization of M-MLV concentration (20, 50 and 200 U per reaction) in WMV-CP (red) and ZYMV-CP (blue) RT-ddPCRs. Lane 1: healthy sample, lane 2: positive sample diluted at 10^{-4}, Lane 3: positive sample diluted at 10^{-3}.
